# Potential roles of hsa_circ_000839 and hsa_circ_0005986 in breast cancer

**DOI:** 10.1002/jcla.24263

**Published:** 2022-01-31

**Authors:** Zahra Firoozi, Elham Mohammadisoleimani, Abbas Shahi, Hosein Mansoori, Mohammad Mehdi Naghizadeh, Milad Bastami, Ziba Nariman‐Saleh‐Fam, Abdolreza Daraei, Atefeh Raoofat, Yaser Mansoori

**Affiliations:** ^1^ Department of Medical Genetics Fasa University of Medical Sciences Fasa Iran; ^2^ Department of Medical Biotechnology Fasa University of Medical Sciences Fasa Iran; ^3^ Department of Immunology School of Medicine Tehran University of Medical Science Tehran Iran; ^4^ Noncommunicable Diseases Research Center Fasa University of Medical Sciences Fasa Iran; ^5^ Women's Reproductive Health Research Center Tabriz University of Medical Sciences Tabriz Iran; ^6^ Department of Medical Genetics School of Medicine Babol University of Medical Sciences Babol Iran; ^7^ 48435 Department of Medical Genetics School of Medicine Shiraz University of Medical Sciences Shiraz Iran

**Keywords:** breast cancer, circRNAs, hsa_circ_0005986, hsa_circ_000839

## Abstract

**Background:**

Breast cancer (BC) is one of the leading causes of death among women around the world. Circular RNAs (circRNAs) are a newly discovered group of non‐coding RNAs that their roles are being investigated in BC and other cancer types. In this study, we evaluated the association of hsa_circ_0005986 and hsa_circ_000839 in tumor and adjacent normal tissues of BC patients with their clinicopathological characteristics.

**Materials and methods:**

Total RNA was extracted from tumors and adjacent non‐tumor tissues by the Trizol isolation reagent, and cDNA was synthesized using First Strand cDNA Synthesis Kit (Thermo Scientific). The expression level of hsa_circ_0005986 and hsa_circ_000839 was quantified using RT‐qPCR. Online in silico tools were used for identifying potentially important competing endogenous RNA (ceRNA) networks of these two circRNAs.

**Results:**

The expression level of hsa_circ_0005986 and hsa_circ_000839 was lower in the tumor as compared to adjacent tissues. The expression level of hsa_circ_0005986 in the patients who had used hair dye in the last 5 years was significantly lower. Moreover, a statistically significant negative correlation between body mass index (BMI) and hsa_circ_000839 expression was observed. In silico analysis of the ceRNA network of these circRNAs revealed mRNAs and miRNAs with crucial roles in BC.

**Conclusion:**

Downregulation of hsa_circ_000839 and hsa_circ_0005986 in BC tumors suggests a tumor‐suppressive role for these circRNAs in BC, meriting the need for more experimentations to delineate the exact mechanism of their involvement in BC pathogenesis.

## Graphical abstract

This study indicated that hsa_circ_000839 and hsa_circ_0005986 have a tumor‐suppressive role and could be involved in the development of BC by regulating ceRNA networks.

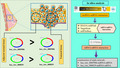

## 1 BACKGROUND

Breast cancer (BC) is the most frequent malignancy among women worldwide. About 70%–80% of patients diagnosed in the early stage will be cured.^1^ Although its mortality has reduced due to the emergence of currently available medications, it is estimated that BC is the leading cause of about 450,000 deaths per year.^2^, ^3^ Various factors affect the progression and response to treatment of BC patients, such as the presence or lack of human epidermal growth factor receptor 2 (HER2/neu), progesterone receptor (PR) or estrogen receptor (ER), and lymph node metastasis as well as tumor size.^2^, ^4^, ^5^ Also, it is revealed that various environmental factors and dysregulation of several genes, contribute to the BC progression and metastasis in different ways.^6^, ^7^, ^8^, ^9^ Recently, it has been shown that non‐coding RNAs, including long non‐coding RNAs (lncRNAs), microRNAs (miRNAs), and circular RNAs (circRNAs) are associated with the pathobiology of breast tumors.^10^, ^11^ Among these, circRNAs are less studied and emerging evidence supports their crucial role in BC tumorigenesis. These most recently discovered ncRNAs are associated with the occurrence and development of different abnormalities such as cardiovascular diseases,^12^, ^13^ central nervous dysfunctions,^14^, ^15^ and different types of cancers.^16^, ^17^

CircRNAs may expose a sponge effect on miRNAs and thereby regulate their target mRNAs. They are also involved in adjusting the activity of RNA binding proteins (RBP) and transcription regulation.^18^, ^19^, ^20^ Due to their stability and abundance in body fluids, circRNAs are promising biomarkers candidates in cancer.^21^, ^22^, ^23^, ^24^ Increasing evidence proposed that circRNAs are involved in proliferation, migration, invasion, and apoptosis of BC.^10^, ^17^, ^25^ For example, hsa_circ_001783 has been demonstrated to enhance the proliferation and invasion of BC by sponging miR‐200c‐3p. Moreover, Liang HF et al. have shown that upregulation of hsa_circ_0008717 can significantly increase the progression and proliferation of BC by sponging miR‐1271.^17^, ^26^

This study set out to investigate the potential contribution of two circRNAs, hsa_circ_0005986 and hsa_circ_000839, in BC pathogenesis. Hsa_circ_0005986, originated from the PRDM2 (PR/SET Domain 2) gene,^27^ is a promising biomarker for hepatocellular carcinoma (HCC), possibly regulating NOTCH1 expression through sponging and inhibiting miR‐129‐5p.^28^ Hsa_circ_000839, also known as hsa_circ_0000497, is a circRNA derived from the SLAIN gene^27^ regulating the expression of miR‐200b in HCC. It has been shown that decreased levels of RhoA and hsa_circ_000839 by mir‐200b inhibited the migration and invasion of HCC cells. This evidence suggests that hsa_circ_000839/miR‐200b/RhoA axis can regulate the invasion and metastasis in HCC.^29^

Besides, microarray studies have shown that hsa_circ_000839 could also be a potential biomarker in multiple myeloma.^30^ Although studies evaluated the potential roles of these circRNAs in a variety of cancers, to the best of our knowledge, the expression pattern and roles of these circRNAs in BC are still unknown. Therefore, this study is aimed at evaluating the expression pattern of hsa_circ_0005986 and hsa_circ_000839 in BC.

## 2 MATERIALS AND METHODS

### 2.1 Patients and tissue sampling

In this study, samples including 50 tumor tissues and 50 adjacent normal tissues were taken from BC patients from Shahid Faghihi hospital, Shiraz, Iran. Patients had not been treated with chemotherapy and radiotherapy before surgery, and samples were collected after taking informed written consent from patients. Tumor and adjacent normal tissues were stored at −80°C after surgery for later use. Clinicopathological and demographic characteristics of patients are provided in Table 1 and Table 2. We categorized patients into two groups based on whether they have used hair dye in the last 5 years ago or not. Also, participants were divided into two groups according to the age at the first full‐term pregnancy of <25 or ≥25 years. The study was approved by the local ethical committee at the Fasa University of Medical Sciences (FUMS).

**TABLE 1:** Relation of hsa_circ_0005986 and hsa_circ_000839 expression with clinicopathological and demographic characteristics in breast cancer patients

		hsa_circ_0005986 level					hsa_circ_000839 level				
		N	Mean	SD	Median	*p*‐value	N	Mean	SD	Median	*p*‐value
Age	<50	22	1.074	3.405	0.179	0.452	22	1.146	1.621	0.738	0.754
	≥50	28	0.416	0.700	0.243		28	1.324	1.805	0.621	
Tumor size	<2.5 cm	31	0.373	0.678	0.191	0.294	31	1.322	1.712	0.794	0.294
	≥2.5 cm	19	1.247	3.646	0.262		19	1.121	1.749	0.438	
Estrogen receptor	Negative	1	0.778	0	0.778	0.136	1	2.383	0	2.383	0.177
	Positive	49	0.704	2.337	0.213		49	1.222	1.721	0.654	
Progesterone receptor	Negative	2	1.923	2.663	1.923	0.882	2	1.143	.867	1.143	0.621
	Positive	48	.655	2.315	0.220		48	1.250	1.743	0.695	
HER2	Negative	33	.947	2.826	0.251	0.223	33	1.303	1.968	0.736	0.838
	Positive	17	0.235	0.222	0.139		17	1.135	1.096	0.587	
Nuclear grade	1	6	0.228	0.201	0.171	0.743	6	.845	0.721	0.592	0.953
	2	36	0.462	0.781	0.243		36	1.381	1.956	0.648	
	3	8	2.158	5.608	0.187		8	.935	0.807	0.738	
Lymph node metastasis	Yes	15	1.471	4.104	0.161	0.907	15	1.145	1.972	0.394	0.144
	No	35	0.377	0.636	0.227		35	1.288	1.617	0.825	
Age at FFTP	<25	31	0.907	2.885	0.251	0.382	31	1.333	1.735	0.654	0.634
	≥25	13	0.254	0.257	0.139		13	0.843	0.647	0.741	
Age at menarche	<14	31	0.818	2.879	0.145	0.108	31	1.303	2.078	0.467	0.255
	≥14	19	0.521	0.830	0.251		19	1.151	0.871	0.974	
Age at menopause	<50	14	0.250	0.242	0.186	0.266	14	0.914	0.642	0.900	0.637
	≥50	21	0.477	0.791	0.262		21	1.538	2.033	0.654	
Number of abortions	0	36	0.869	2.715	0.202	0.430	36	1.274	1.942	0.641	0.136
	1	12	0.317	0.230	0.243		12	1.321	0.931	1.102	
	>1	2	0.097	0.056	0.097		2	0.268	0.019	0.268	
Hair dye use	No	10	1.992	4.940	0.437	0.012	10	1.193	0.854	1.061	0.344
	Yes	40	0.384	0.747	0.142		40	1.259	1.874	0.641	

Abbreviations: FFTP, first full‐term pregnancy; IDC, invasive ductal carcinoma; ILC, invasive lobular carcinoma.

**TABLE 2:** Relation of hsa_circ_0005986 and hsa_circ_000839 expression with clinicopathological characteristics in breast cancer patients, according to dividing patients into two groups of high and low expressions

		hsa_circ_0005986 level					hsa_circ_000839 level				
		Low		High		*p*‐value	Low		High		*p*‐value
		N	%	N	%		N	%	N	%	
Age	<50	12	54.5%	10	45.5%	0.569	10	45.5%	12	54.5%	0.569
	≥50	13	46.4%	15	53.6%		15	53.6%	13	46.4%	
Menopausal status	Pre	9	60.0%	6	40.0%	0.355	19	61.3%	12	38.7%	0.355
	Post	16	45.7%	19	54.3%		6	31.6%	13	68.4%	
Age at menopause	<50	7	50.0%	7	50.0%	0.679	9	60.0%	6	40.0%	0.332
	≥50	9	42.9%	12	57.1%		16	45.7%	19	54.3%	
BMI	≤25	9	45.0%	11	55.0%	0.826	5	35.7%	9	64.3%	0.047
	25–29	12	54.5%	10	45.5%		11	52.4%	10	47.6%	
	≥30	4	50.0%	4	50.0%		6	30.0%	14	70.0%	
Age at FFTP	<25	13	41.9%	18	58.1%	0.099	15	68.2%	7	31.8%	0.741
	≥25	9	69.2%	4	30.8%		4	50.0%	4	50.0%	
Breastfeeding duration	0	3	50.0%	3	50.0%	0.811	16	51.6%	15	48.4%	0.811
	<24	8	57.1%	6	42.9%		6	46.2%	7	53.8%	
	≥24	14	46.7%	16	53.3%		3	50.0%	3	50.0%	
Abortion history	No	19	52.8%	17	47.2%	0.529	6	42.9%	8	57.1%	0.208
	Yes	6	42.9%	8	57.1%		16	53.3%	14	46.7%	
Tumor size	<2.5 cm	17	54.8%	14	45.2%	0.382	20	55.6%	16	44.4%	0.382
	≥2.5 cm	8	42.1%	11	57.9%		5	35.7%	9	64.3%	
Estrogen receptor	Negative	0	0.0%	1	100.0%	0.312	14	45.2%	17	54.8%	0.312
	Positive	25	51.0%	24	49.0%		11	57.9%	8	42.1%	
Progesterone receptor	Negative	1	50.0%	1	50.0%	1.000	0	0.0%	1	100.0%	1.000
	Positive	24	50.0%	24	50.0%		25	51.0%	24	49.0%	
Her2	Negative	15	45.5%	18	54.5%	0.370	1	50.0%	1	50.0%	0.765
	Positive	10	58.8%	7	41.2%		24	50.0%	24	50.0%	

Abbreviations: BMI, body mass index; FFTP, first full‐term pregnancy.

### 2.2 Extraction of total RNA and cDNA synthesis

Total RNA was extracted from tumors and adjacent non‐tumor tissues by the Trizol isolation reagent (Invitrogen, Thermo Fisher) according to the manufacturer's instructions. RNA integrity was examined by gel electrophoresis, and the absorption ratio at 260–280 nm (A260/280) was used for assessing the quantity of RNA. First Strand cDNA Synthesis Kit (Fermentas, Cat. No: K1622) was used for cDNA synthesis according to the manufacturer's instructions.

### 2.3 Real‐time PCR

The real‐time PCR assay was performed using 10 µl RealQ Plus 2x Master Mix Green with High ROX (Ampliqon, Cat. No: A325402‐25), along with 2 µl of cDNA, 1 µl of each primer, and 6 µl DNase‐free dH2O for per 20 µl reaction volume. circRNAs sequences were obtained from CircInteractome.^31^ Specific primer sequences are shown in Table 3. The relative expression was calculated by the $2^{‐\Delta \Delta C_{T}}$ (fold change) method.

**TABLE 3:** Primers sequences

CircRNAs and housekeeping gene	Primer	
hsa_circ_0005986	Forward	CTATAAAACTTTAAAGAACACTACTGAGCC
	Reverse	GGGTCTTGTCAACAGCAGAA
hsa_circ_000839	Forward	CAATCTTGTAGTTATACATTGGC
	Reverse	AGTCTAAAGCACAGGGAAC
B2 M (housekeeping gene)	Forward	AGATGAGTATGCCTGCCGTG
	Reverse	GCGGCATCTTCAAACCTCCA

### 2.4 Statistical analysis

Data analyses have been carried out by IBM SPSS 21, and *p*‐value <0.05 was considered the statistically significant, and graphical presentation was done using GraphPad Prism 6.0.1 and MedCalc 18.2.1. Wilcoxon test was applied to compare circRNAs expression among tumors and adjacent normal tissues. The association of circRNAs expression with demographic and clinicopathological features of BC patients was assessed by Mann–Whitney and Kruskal–Wallis tests. Samples were categorized into groups of high and low expressions based on the median fold changes of each gene. Differences between these groups in terms of demographic variables were analyzed using either a chi‐square test or an independent t test.

### 2.5 Construction of the ceRNA regulatory network

Circinteractome (https://circinteractome.nia.nih.gov) database was used to predict interactions between circRNAs and miRNAs, whereas DIANA‐miRPath (http://diana.imis.athena‐innovation.gr/DianaTools/index.php?r=mirpath), TargetScan (http://www.targetscan.org/vert_72/), and mirTarBase (http://miRTarBase.mbc.nctu.edu.tw/) were used to predict interactions between miRNAs and mRNAs.^31^, ^32^, ^33^, ^34^ The miRNA‐mRNA pairs were searched in three databases, and in the next step, interactions with validation methods and strong evidence were selected. Using this approach, we identified circRNA‐miRNA and miRNA‐mRNA pairs. In addition, we examined PPI (protein‐protein interaction) using the STRING database (https://string‐db.org).^35^ The data were visualized using Cytoscape software (version 3.7.2).^36^

To strengthen the data of hsa_circ_000839 and hsa_circ_0005986 networks, we analyzed the expression of mRNAs and miRNAs observed in these networks in TCGA‐BRCA using the TACCO database (http://tacco.life.nctu.edu.tw/).^37^ Then, we investigated the possible significant correlation between these mRNAs and miRNAs in these networks. Using Enrichr (https://maayanlab.cloud/Enrichr/), then we analyzed the biological processes (BP), molecular function (MF), and KEGG pathways for the genes with the highest significant correlation to miRNAs.^38^

### 2.6 Survival analysis

To evaluate the importance of mRNAs in the ceRNA network of these circRNAs, we set out to evaluate the association of mRNAs with the survival of BC patients in publicly available datasets. The measure of overall survival would compare the number of patients who had died and not died related to these genes in BRCA. To this end, we used the GEPIA database which performs log‐rank survival analysis using TCGA data. GEPIA database uses a log‐rank test for statistical analysis based on gene expression.^39^ The threshold to investigate the importance of survival was log‐rank *p *< 0.05.

## 3 RESULTS

### 3.1 The expression level of hsa_circ_0005986 and hsa_circ_000839 in tumors and paired tumor‐adjacent tissues

**FIGURE 1:**
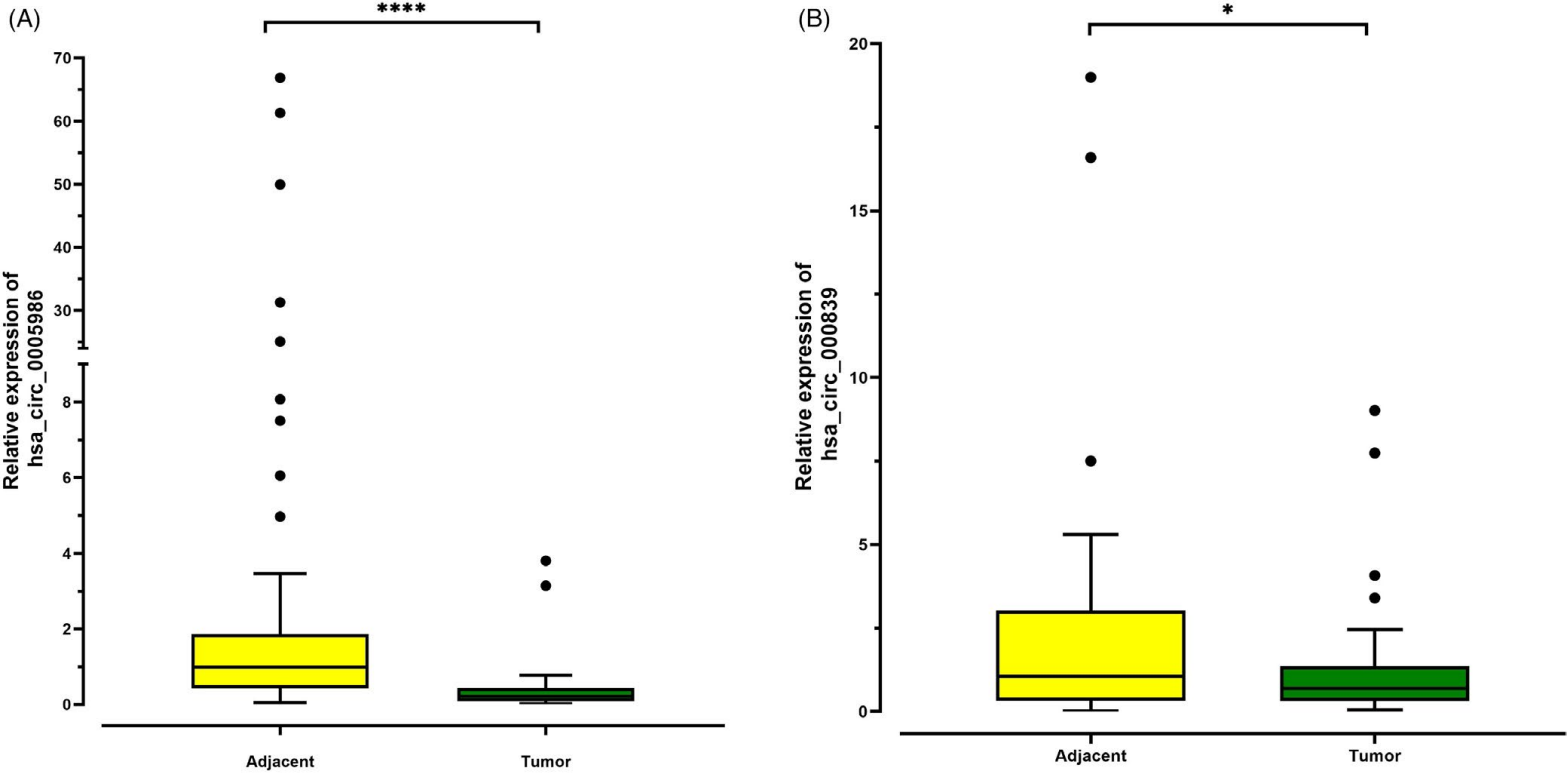
Box and whisker plot (Tukey style, outliers in black dots) of the expression of hsa_circ_0005986 and hsa_circ_000839 in tumors and paired non‐cancerous tissues. (A, B) Down expression of both hsa_circ_0005986 and hsa_circ_000839 in tumors compared with adjacent normal tissues (*p ≤ 0.05; **p ≤ 0.01; ***p ≤ 0.001; ****p ≤ 0.0001)

The evaluation of circRNAs expression by RT‐qRCR method in 50 tumor samples and 50 tumor's adjacent normal tissues showed that hsa_circ_0005986 had significantly lower expression level in tumor tissues (Median = 0.220, IQR = 0.362) compare to the adjacent normal tissues (Median = 0.987, IQR = 1.43; *p*‐value < 0.001, Figure 1A). Also, the expression level of hsa_circ_000839 was significantly lower in tumors tissues (Median = 0.695, IQR = 1.05) in comparison to the tumor's adjacent normal tissues (Median = 1.055, IQR = 2.69; *p*‐value = 0.022, Figure 1B).

### 3.2 Relation of hsa_circ_0005986 expression with clinicopathological and demographic characteristics in breast cancer patients

**FIGURE 2:**
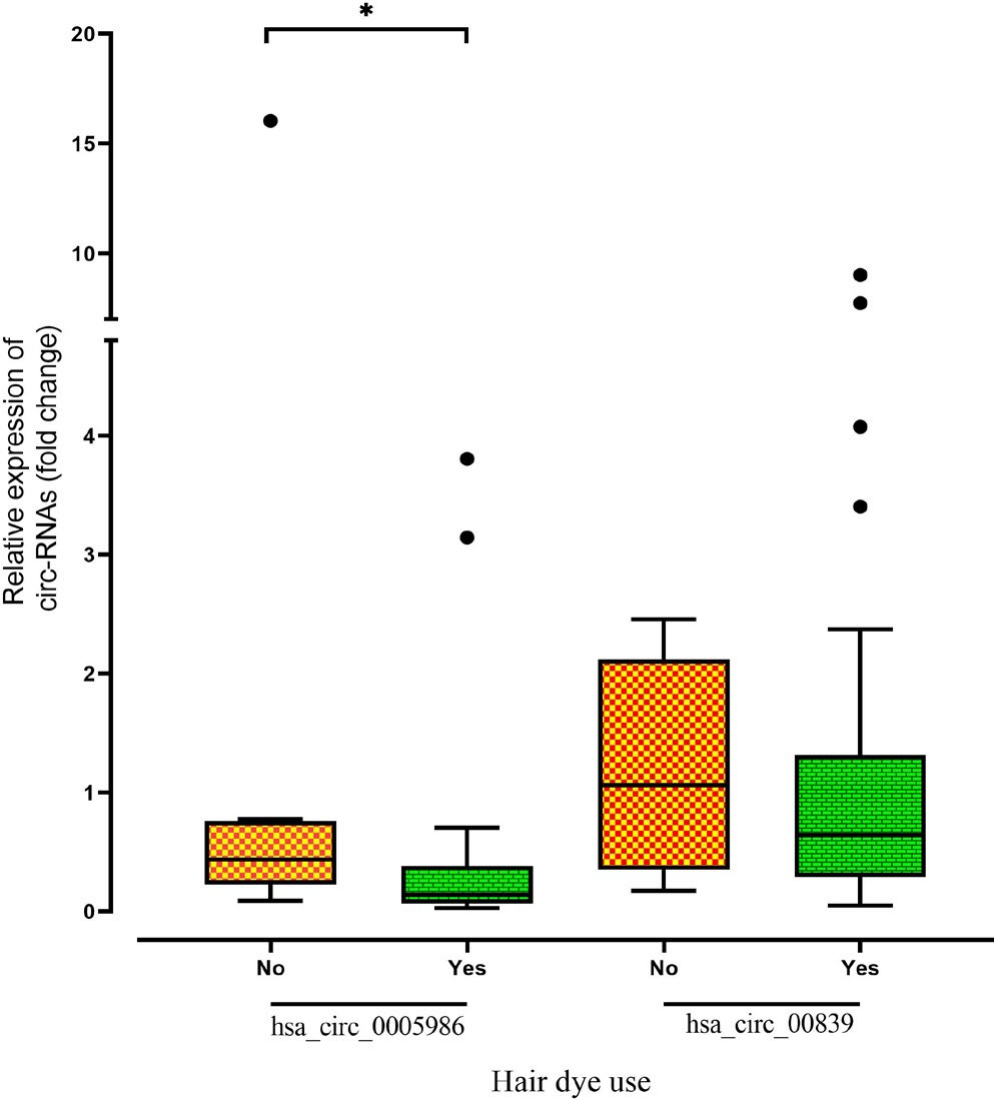
Box and whisker plot (Tukey style, outliers in black dots) of the expression level of hsa_circ_0005986 and hsa_circ_000839 in hair dye users in the last 5 years (*n* = 40) and non‐users in this time period (*n* = 10) (*p ≤ 0.05; **p ≤ 0.01; ***p ≤ 0.001; ****p ≤ 0.0001)

Data analysis revealed that the expression level of hsa_circ_0005986 was significantly lower in patients who have used hair dye in the last 5 years than patients who have not used hair dye in this time period (*p*‐value = 0.012, Figure 2). Furthermore, according to the median for hsa_circ_0005986 expression level, BC patients were divided into high and low expression groups (Table 2). The comparison of clinicopathological and demographic data between low and high expression groups did not show any significant difference.

### 3.3 Relation of hsa_circ_000839 expression with clinicopathological and demographic characteristics in breast cancer patients

**FIGURE 3:**
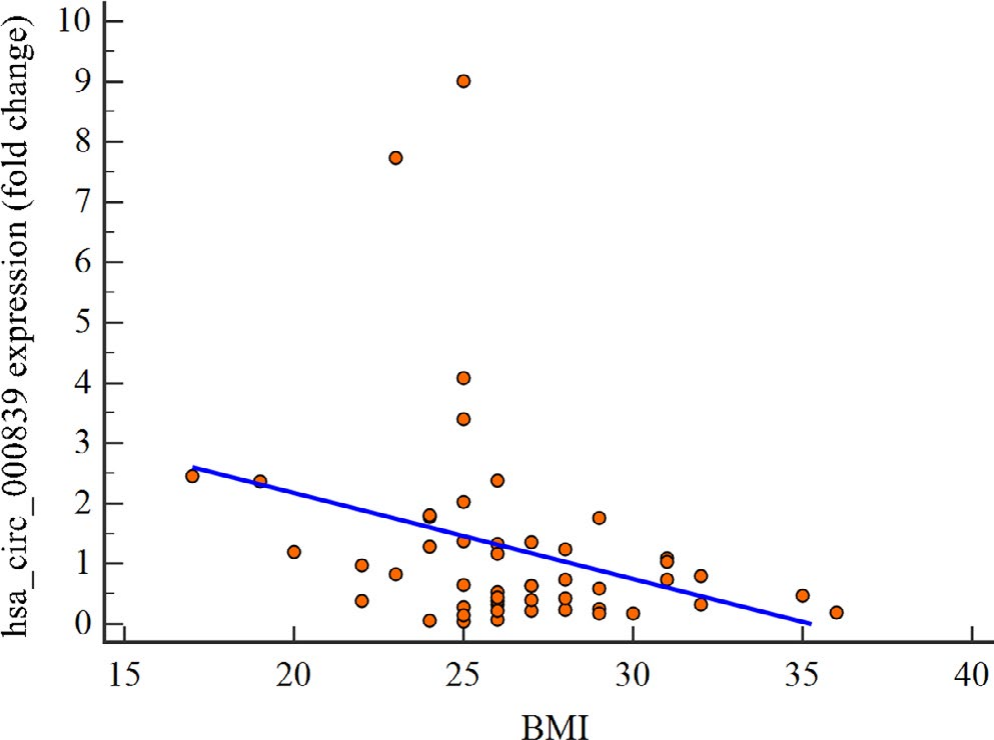
Correlation between BMI and expression levels of hsa_circ_000839 (r = −0.357, *p*‐value = 0.01)

Comparison of hsa_circ_000839 expression level between patients who had used hair dye in the last 5 years with those who had not used hair dye in this period of time did not show a significant difference (*p*‐value = 0.344, Figure 2), but the correlation analysis revealed that hsa_circ_000839 expression level has a significant negative correlation with body mass index (BMI) (r = −0.357, *p*‐value = 0.01, Figure 3). Similar to hsa_circ_0005986, BC patients were divided into high and low expression groups (Table 2). There was not any significant difference between clinicopathological and demographic data in low and high hsa_circ_000839 expression groups except in the BMI case which could affect the expression of hsa_circ_000839.

### 3.4 Potential circRNAs‐mediated sponge regulatory network

**FIGURE 4:**
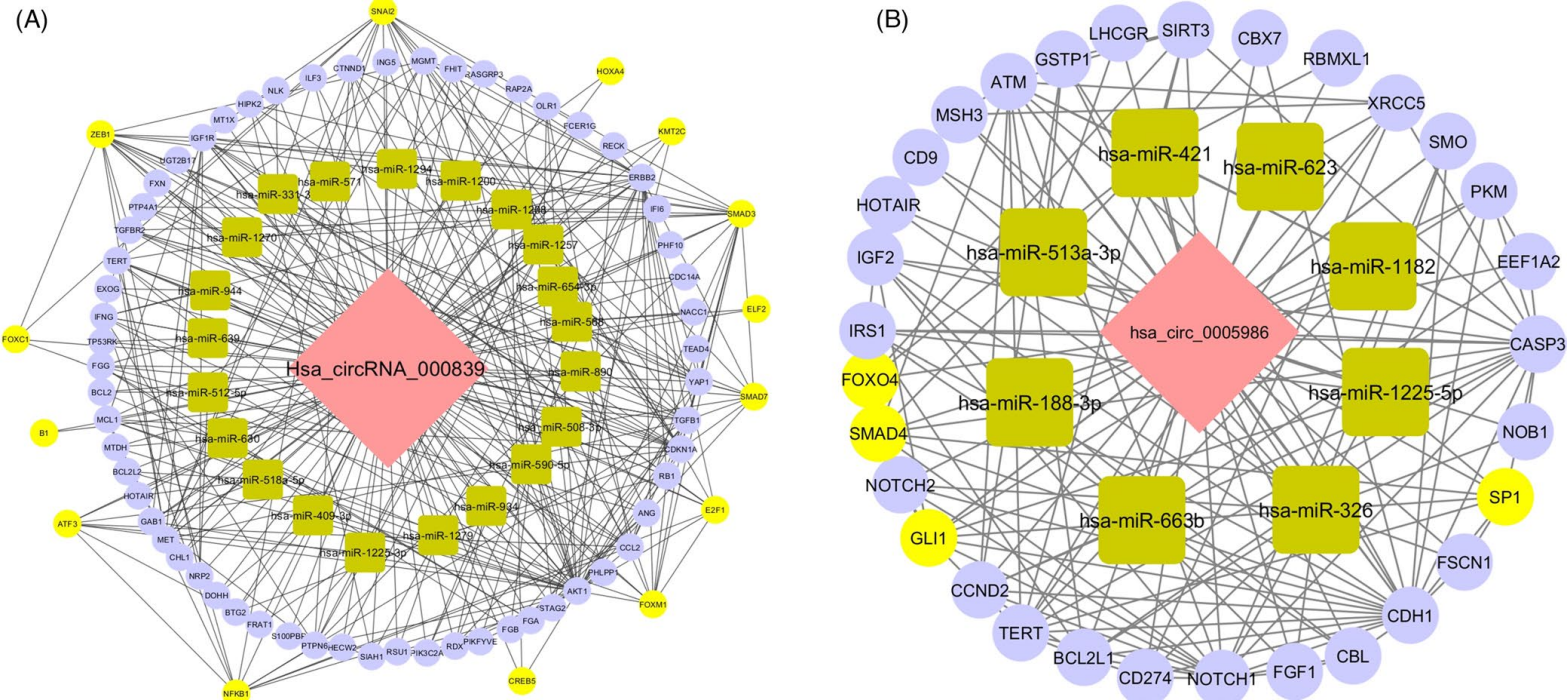
The triple regulatory networks of circRNA‐miRNA‐mRNA were constructed based on circRNA‐miRNA pairs, miRNA‐mRNA pairs, and PPI. Pink indicates circRNAs, jade indicates miRNAs, and purple indicates mRNAs. The yellow mRNAs play the role as a transcription factor. In order to evaluate the genes with transcription factor role, we used TF2DNA (http://fiserlab.org/tf2dna_db//search_genes.html) database. A and B are ceRNA network hsa_circ_000839 and hsa_circ_0005986, respectively

The hsa_circ_000839/miRNA/mRNA and hsa_circ_0005986/miRNA/mRNA regulatory networks were constructed separately by in silico investigation. The hsa_circ_000839/miRNA/mRNA network was constructed based on 1 circRNA, 21 miRNAs, and 73 mRNAs (Figure 4A). We identified 21 hsa_circ_000839/miRNA interaction pairs and 73 miRNA/mRNA interaction pairs and 185 protein‐protein interaction (PPI) pairs. The SMAD3, ZEB1, SNAI2, FOXM1, NFKB1, SMAD7, E2F1, ATF3, and hsa‐mir‐1200 had the most interaction among the mRNAs and miRNA in this network, respectively. The hsa_circ_0005986/miRNA/mRNA based on 1 circRNA network was constructed, 8 miRNAs, and 31 mRNAs (Figure 4B). The GLI1, SMAD4, SP1, and FOXO4 had the most interactions among the mRNAs in this network as well as hsa‐mir‐326 and hsa‐mir‐421 with the most interactions among the miRNAs.

**FIGURE 5:**
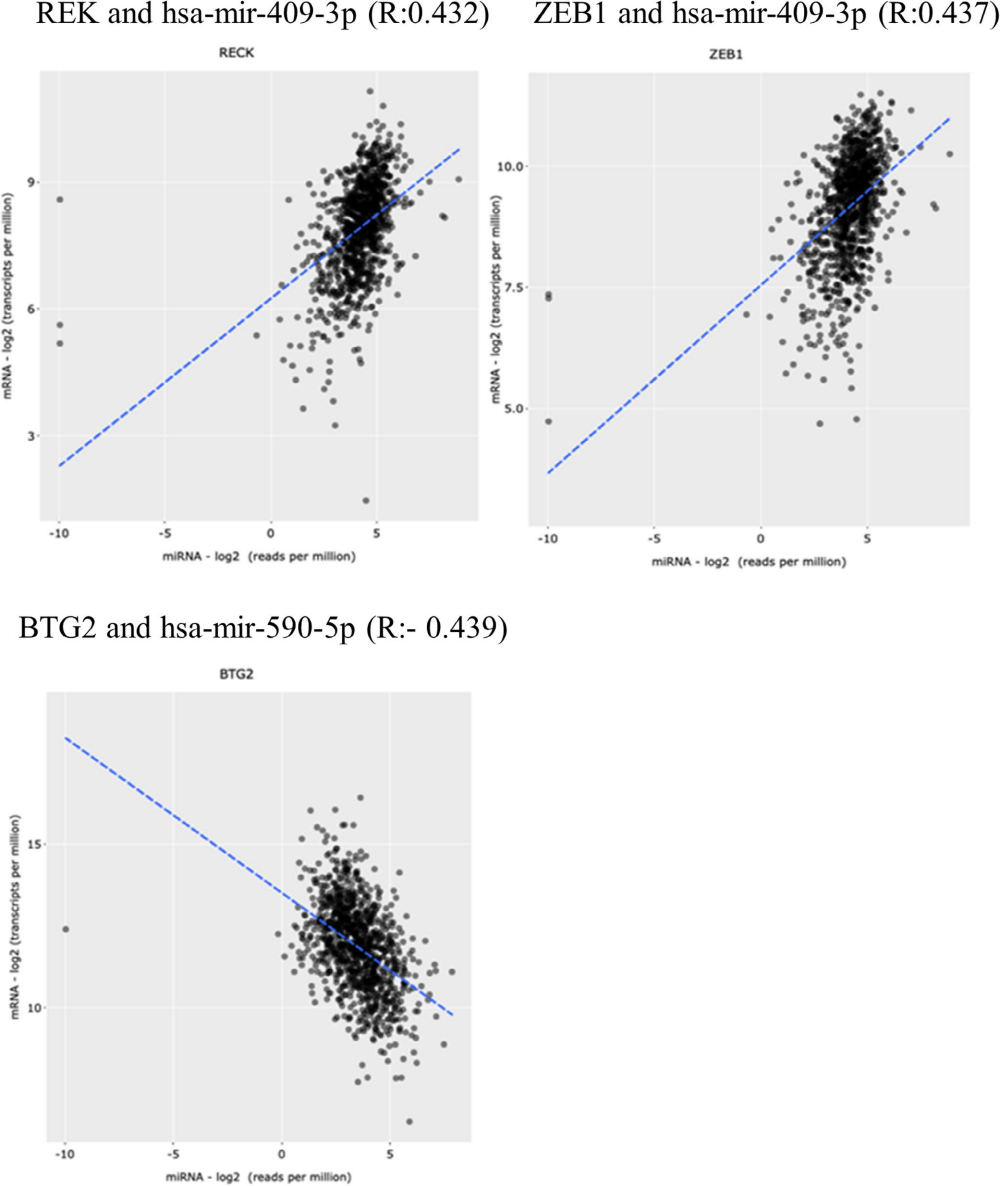
The Most significant correlation between mRNAs and miRNAs in the hsa_circ_000839 network

**FIGURE 6:**
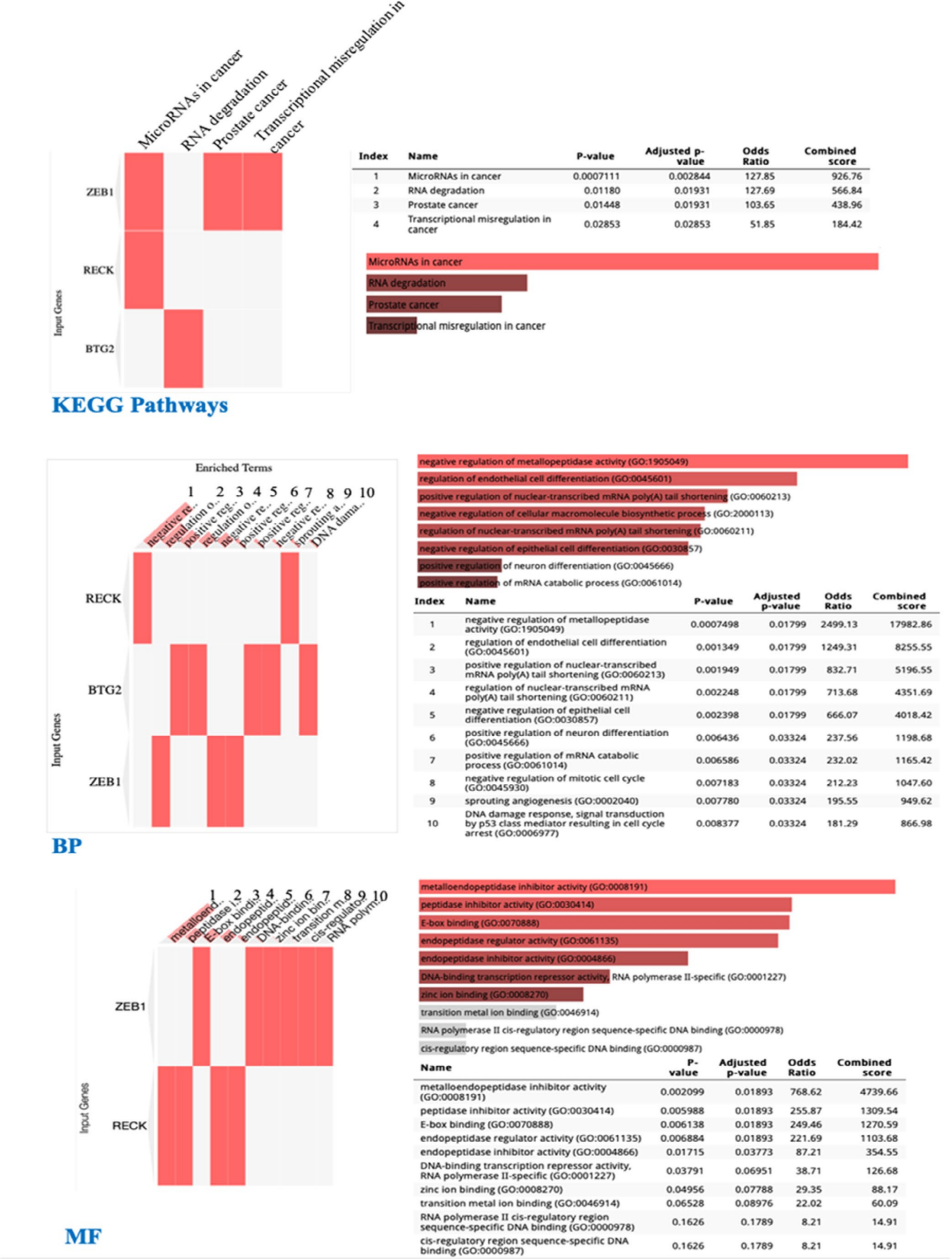
Data extracted from Enrichr for GO terms of genes with the highest significant correlation to miRNAs of hsa_circ_000839 network

Regarding the hsa_circ_000839 network, we found the lower expression of hsa‐mir‐654‐3p, hsa‐mir‐409‐3p, and hsa‐mir‐944 and higher expression of hsa‐mir‐331‐3p and hsa‐mir‐590‐5p (Table 4) in TCGA‐BRCA. Among the mRNAs in this network, we observed altered expression of several genes given in Table 5. Additionally, some of these mRNAs showed positive or negative significant correlations, from very weak to fairly strong, to the above‐mentioned miRNAs (Pearson's correlation coefficient (R) was considered: 0.00–0.19 as very weak, 0.20–0.39 as weak, 0.40–0.59 as fairly strong^40^). Strong positive correlations were seen between RECK and hsa‐mir‐409‐3p (R: 0.432) and also ZEB1 and hsa‐mir‐409‐3p (R: 0.437). On the other hand, a fairly strong negative correlation was seen between BTG2 and hsa‐mir‐590‐5p (R: −0.439) (Figure 5). All information related to the correlation between all mRNAs and miRNAs in this network is provided in Appendix S1. These data might indicate the possible major role of these miRNAs and mRNAs in the hsa_circ_000839 network and BC pathogenesis. The KEGG, the BP, and the MF GO (Gene Ontology) terms for these genes are also given in Figure 6. These GO terms data show their involvement in cancer pathogenesis and pathways. The KEGG pathway data showed the involvement of ZEB1 and RECK in microRNA networks in cancer. The ZEB1 is also involved in prostate cancer and transcriptional misregulation in cancer. Regarding BTG2, it is involved in RNA degradation. The most significant BP related to the RECK gene is “negative regulation of metallopeptidase activity” (GO: 1905049) (*p*‐value: 0.0002500). For MF GO terms, metalloendopeptidase inhibitor activity (GO: 0008191) (RECK gene) was the most significant term. All KEGG, BP, and MF GO terms information for these genes are also given in Figure 6.

**TABLE 4:** Expression of miRNAs involved in hsa_circ_000839 network in TCGA‐BRCA

TCGA‐Breast Cancer	Fold change	Log2 (fold change)	Mean RPM (Tumor)	Mean RPM (Normal)	*p*‐value	Adjusted *p*‐value
Hsa‐mir−654‐3p	−2.11	−1.08	21.02	44.44	2.73e−27	1.36e−26
Hsa‐mir−331‐3p	1.99	1	33.08	16.59	1.45e−16	4.14e−16
Hsa‐mir−409‐3p	−1.22	−0.28	14.37	17.48	0.0308	0.0383
Hsa‐mir−590‐5p	2.07	1.05	14.23	6.87	6.18e−21	2.14e−20
Hsa‐mir−944	−2.95	−1.56	1.9	5.63	1.9e−18	5.91e−18

**TABLE 5:** Expression of mRNAs involved in hsa_circ_000839 network in TCGA‐BRCA

TCGA‐Breast Cancer	Fold change	Log2 (fold change)	Mean RPM (Tumor)	Mean RPM (Normal)	*p*‐value	Adjusted *p*‐value
E2F1	6.69	2.74	481.58	72.02	7.25e−55	2.49e−53
DOHH	1.92	0.94	249.61	130.1	2.23e−18	6.34e−18
PHLPP1	−1.53	−0.61	529.71	810.35	4.01e−29	1.92e−28
NACC1	2.68	1.42	2245.15	837.78	4.47e−60	4.58e−58
STAG2	−1.28	−0.35	2660.04	3396.02	4.19e−18	1.18e−17
RSU1	−1.19	−0.25	1704.75	2031.11	7.37e−14	1.69e−13
NLK	1.59	0.67	620.47	389.92	3.24e−20	1,00E−19
AKT1	1.56	0.64	4685.47	3005.53	1.9e−24	7.21e−24
ELF2	−1.22	−0.28	823.11	1000.64	1.79e−17	4.88e−17
CDKN1A	−1.37	−0.45	2089.83	2859.49	0.00108	0.00146
TGFBR2	−4.77	−2.25	2180.65	10397.43	3.02e−61	4.74e−59
CREB5	−4.43	−2.15	120.84	535.15	3.37e−55	1.21e−53
SMAD3	−1.38	−0.46	1253.11	1729.32	3.32e−22	1.13e−21
TGFB1	1.79	0.84	1126.35	627.71	6.16e−22	2.07e−21
ILF3	1.34	0.42	5725.06	4284.99	3.19e−26	1.31e−25
SMAD7	−1.08	−0.11	701.9	756.21	0.0167	0.0207
EXOG	−1.15	−0.2	112.16	129.2	2.25e−9	4.16e−9
HECW2	−1.14	−0.19	243.81	278.16	0.00733	0.00932
ERBB2	3.77	1.91	18327.05	4862.24	1.09e−15	2.72e−15
HOTAIR	6.81	2.77	131.75	19.36	9.82e−17	2.58e−16
NACC1	2.68	1.42	2245.15	837.78	4.47e−60	4.58e−58
ING5	−1.23	−0.3	400.95	493.68	4.19e−12	8.86e−12
FGB	6.8	2.77	318.5	46.8	0.0000614	0.0000897
FGG	9.43	3.24	252.52	26.78	5.27e−7	8.67e−7
PHF10	−1.55	−0.64	1347.96	2094.1	1.92e−37	1.48e−36
FRAT1	1.2	0.26	150.2	125.43	0.0112	0.0141
RASGRP3	−1.23	−0.3	284.3	349.7	5.14e−10	9.82e−10
NLK	1.59	0.67	620.47	389.92	3.24e−20	1,00E−19
AKT1	1.56	0.64	4685.47	3005.53	1.9e−24	7.21e−24
ZEB1	−2.18	−1.13	721.94	1575.72	7.33e−21	2.35e−20
GAB1	−1.4	−0.49	806.3	1132.79	1.82e−20	5.72e−20
ATF3	−4.31	−2.11	908.78	3914.2	3.87e−39	3.3e−38
OLR1	4.53	2.18	396.82	87.51	2.36e−42	2.48e−41
RB1	−1.2	−0.26	1146.87	1373.79	6.41e−8	1.1e−7
BTG2	−1.56	−0.64	5579.14	8695.09	1.6e−13	3.62e−13
SIAH1	−1.06	−0.09	589.32	627.36	3.65e−7	6.06e−7
CTNND1	−1.05	−0.07	8594.33	9050.41	0.0000029	0.0000046
RDX	−1.56	−0.64	1278.52	1998.39	1.06e−20	3.35e−20
MET	−2.96	−1.57	633.16	1876.17	2.49e−51	5.59e−50
ANG	−1.55	−0.63	328.72	510.24	5.09e−19	1.49e−18
NRP2	−1.15	−0.21	1054.3	1215.51	0.0000667	0.0000972
CTNND1	−1.05	−0.07	8594.33	9050.41	0.0000029	0.0000046
CHL1	−8.74	−3.13	167.04	1459.62	4.3e−61	6.28e−59
RECK	−3.58	−1.84	307.88	1102.33	4.14e−52	1.01e−50

Concerning the hsa_circ_0005986, our data revealed the significant upregulation of hsa‐mir‐421 and hsa‐mir‐188‐3p and downregulation of hsa‐mir‐326 in TCGA‐BRCA (Table 6). Moreover, several mRNAs of this network showed altered expression in TCGA‐BRCA (Table 7). In this network, then we investigated the correlation between mRNAs and miRNAs and found several significant correlations between them. However, most of these correlations were very weak (Appendix S2). Therefore, our data of expression profile and correlation analysis of mRNAs and miRNAs in hsa_circ_000839 and hsa_circ_0005986 indicated the more important role of hsa_circ_000839 in BC with the most important mRNA and miRNAs as follow: *RECK*, *ZEB1*, and *BTG2* as mRNAs; and hsa‐mir‐409‐3p and hsa‐mir‐590‐5p as miRNAs.

**TABLE 6:** Expression of miRNAs involved in hsa_circ_0005986 in TCGA‐BRCA

TCGA‐Breast Cancer	Fold change	Log2 (fold change)	Mean RPM (Tumor)	Mean RPM (Normal)	*p*‐value	Adjusted *p*‐value
Hsa‐mir−421	1.76	0.82	2.11	1.19	1.63e−8	3.05e−8
Hsa‐mir−326	−2.48	−1.31	9.96	24.71	3.09e−27	1.49e−26
Hsa‐mir−188‐3p	2.1	1.07	1.16	0.55	3.04e−7	5.29e−7

**TABLE 7:** Expression of mRNAs involved in hsa_circ_0005986 in TCGA‐BRCA

TCGA‐Breast Cancer	Fold change	Log2 (fold change)	Mean RPM (Tumor)	Mean RPM (Normal)	*p*‐value	Adjusted *p*‐value
SIRT3	−1.02	−0.02	763.24	775.43	0.0122	0.0153
CBX7	−3.04	−1.6	690.29	2099.51	3.12e−58	1.86e−56
RBMXL1	−1.46	−0.54	580.06	845.87	4.56e−34	2.83e−33
XRCC5	1.18	0.24	8153.2	6881.78	3.62e−12	7.68e−12
SMO	1.1	0.14	554.54	504.59	0.00151	0.00201
PKM	1.85	0.89	22562.44	12187.92	5.1e−41	4.91e−40
EEF1A2	2.58	1.37	2173.55	841.37	9.06e−21	2.89e−20
CASP3	1.6	0.68	862.2	538.15	1.27e−39	1.11e−38
NOB1	−1.36	−0.44	886.26	1206.14	2.72e−21	8.88e−21
SP1	−1.23	−0.3	3199.05	3925.36	6.84e−18	1.9e−17
CDH1	1.46	0.55	10971.64	7496.56	0.000409	1.46
CBL	−1.47	−0.55	839.15	1230.84	1.35e−24	5.16e−24
FGF1	−4.44	−2.15	278.02	1233.99	1.96e−59	1.54e−57
NOTCH1	−1.35	−0.43	1200.8	1621.72	3.36e−19	9.93e−19
CD274	1.17	0.23	33.03	28.22	0.00541	0.00695
BCL2L1	1.41	0.49	2755.09	1958.27	3.05e−22	1.04e−21
TERT	9.04	3.18	7.5	0.83	5.97e−23	2.1e−22
CCND2	−2.12	−1.08	779.73	1649.3	1.91e−47	2.95e−46
GLI1	−1.64	−0.71	28.21	46.3	2.16e−16	5.58e−16
NOTCH2	−1.31	−0.39	5893.17	7713.57	6.23e−14	1.43e−13
SMAD4	−1.48	−0.57	1503.76	2226.52	6.82e−39	5.75e−38
FOXO4	−2.04	−1.03	493.83	1006.57	9,00E−49	1.59e−47
IRS1	−1.66	−0.73	1391.9	2304.28	1.06e−24	4.06e−24
IGF2	−1.45	−0.54	4130.7	5989.32	3.79e−36	2.67e−35
HOTAIR	6.81	2.77	131.75	19.36	9.82e−17	2.58e−16
CD9	1.89	0.92	8784.09	4650.33	9.16e−32	5.06e−31
MSH3	−1.19	−0.26	512.25	612.13	1.94e−8	3.43e−8
ATM	−1.55	−0.63	997.22	1542.7	2.22e−23	8.01e−23
GSTP1	−1.15	−0.21	3603.87	4157.3	7.78e−13	1.7e−12

### 3.5 Survival analysis

**FIGURE 7:**
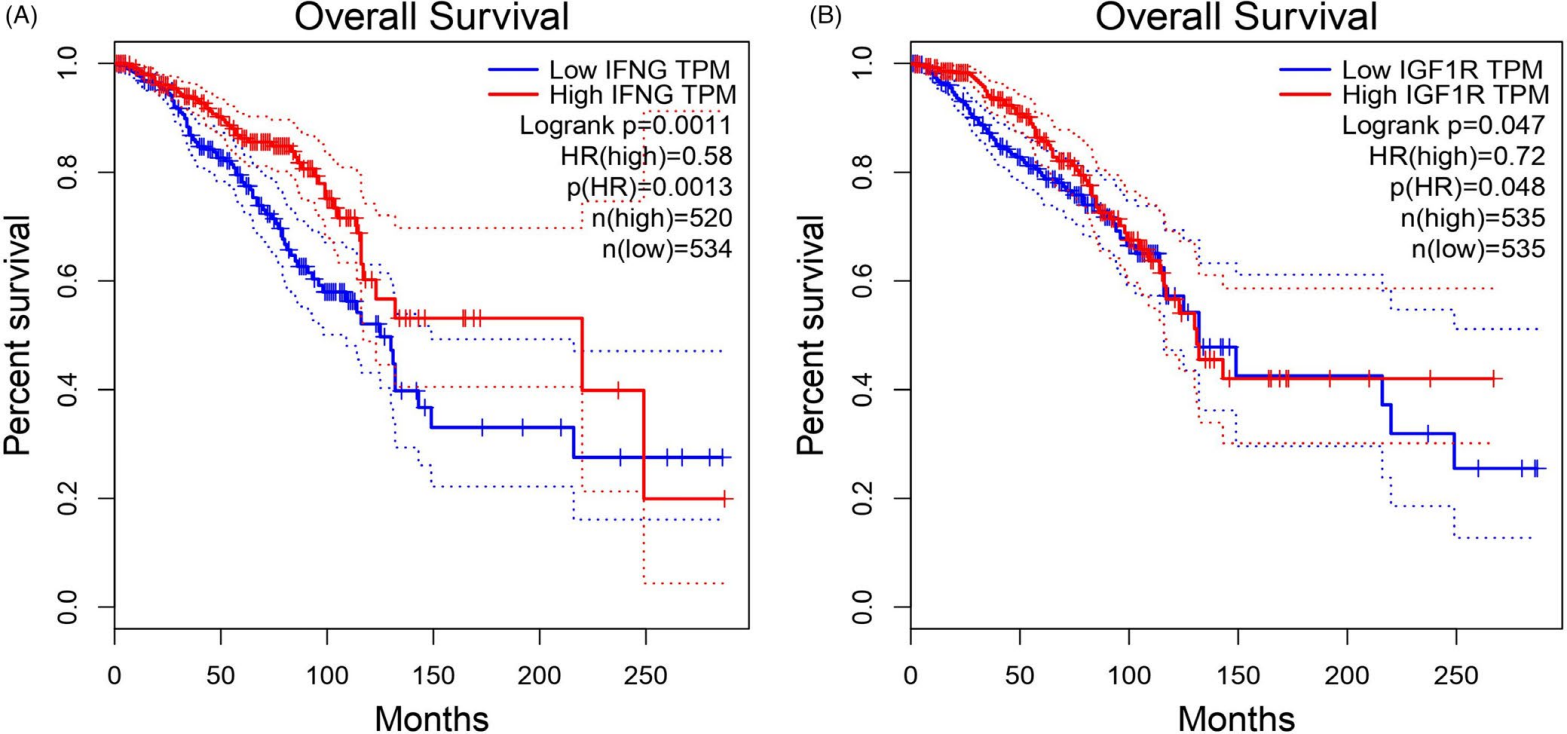
Association of IFNG and IGF1R expression with patient's survival using TCGA dataset in BRCA. High expression of two genes was associated with higher survival. (A) Log‐rank p related to IFNG was 0.0011. (B) Log‐rank p related to IGF1R was 0.047

We performed survival analysis for 73 mRNAs in the hsa_circ_000839/miRNA/mRNA network. Two mRNAs (IFNG and IGF1R) were significantly associated with survival prognosis in patients with BC in the TCGA‐BRCA dataset (Figure 7). In the hsa_circ_0005986/miRNA/mRNA network, no mRNA was significantly associated with survival prognosis in patients with BC.

## 4 DISCUSSION

During the last decade, different studies have focused on the role of circRNAs in the pathophysiology of diverse diseases. As mentioned previously, circRNAs can act as the miRNA sponge element and also, they can bind to RBP, or they can modulate the transcription of target genes. Increasing shreds of evidence propose the vital roles of circRNAs in tumorigenesis, metastasis, chemoresistance, and progression of BC. Besides, some other investigations have unveiled that circRNAs can be considered as appropriate prognostic biomarkers in BC.^10^

In this study, we compared the hsa_circ_000839 and hsa_circ_0005986 expression levels in tumor and adjacent normal tissues in breast cancer patients and their association with patients' clinicopathological and demographic characteristics. To the best of our knowledge, this is the first study that evaluates the association of these two circRNAs in the BC context. In addition, we constructed the ceRNA regulatory network of these circRNAs based on online databases to better figure out their importance in BC circumstances.

Our results show that the expression level of hsa_circ_000839 is significantly lower in tumor tissues than in normal adjacent ones (Figure 1B). There are few investigations about the role of this circRNA in the development and progression of cancers. Zhou et al.^30^ performed a study to investigate the heterogeneity of circRNA expression patterns in multiple myeloma (MM) patients. In the first stage of their study, they used microarray technology to evaluate circRNA expression profiles in MM patients. In this stage, the hsa_circ_000839 came under the top ten downregulated circRNAs in MM, but at the second stage which qPCR was used for validation of top ten up‐ and downregulated circRNAs, the expression level of hsa_circ_000839 did not show a significant difference in MM samples compared with healthy controls (HCs), also it had no correlation with progression‐free survival (PFS) and overall survival (OS) of MM patients. In another study, Wang et al.^41^ have shown that the expression level of hsa_circ_000839 in HCC cells is significantly higher than in normal adjacent tissues. In the following, they found that RhoA (Ras homolog A) as one of the most important factors in invasion and metastasis of HCC has a positive correlation with hsa_circ_000839. While miR‐200b had a negative correlation with both RhoA and hsa_circ_000839. Their functional studies revealed that decreasing the expression of RhoA and hsa_circ_000839 by miR‐200b can inhibit the migration and invasion of HCC. These findings suggest that hsa_circ_000839/miR‐200b/RhoA endogenous competition network may be considered as the potential target for developing new anti‐cancer medications.

Besides, several studies have shown that obesity can be considered as the risk factor for different cancers such as breast cancer,^42^ colorectal cancer,^43^ kidney cancer,^44^ etc. Therefore, we decided to evaluate the correlation between body mass index (BMI) and expression levels of hsa_circ_000839 and hsa_circ_0005986 in BC patients. Our correlation analysis shows a significant negative correlation between the expression level of hsa_circ_000839 in tumor tissues and BMI, but we could not find any significant association between this circRNA and other clinicopathological characteristics. In contrast with hsa_circ_000839, the hsa_circ_0005986 did not show a significant correlation with BMI. As it is obvious the results of studies about the roles of hsa_circ_000839 in the pathobiology of cancers, especially BC are limited and controversial which increases the necessity of more investigation about the role of this circRNA in different tumoral conditions especially BC.

Also, similar to hsa_circ_000839 the expression level of hsa_circ_0005986 was significantly lower in tumor specimens in comparison to adjacent normal tissues (Figure 1A). In line with our results, in a study by Fu et al., the expression level of hsa_circ_0005986 was significantly lower in HCC primary cells and HCC cell lines. They found that the hsa_circ_0005986 can act as a sponge for miR‐129‐5p and its downregulation leads to an increase of miR‐129‐5p and NOTCH1 expression levels which subsequently result in enhancement of cell proliferation and transition of the cell cycle from G0/G1 to G2 phase. These events suggest that this circRNA may have a vital role in the progression and development of cancer situation. Nevertheless, there is little evidence about the roles of this circRNA in BC and other types of cancers, and more investigations are needed to elucidate its exact roles in cancers.

Also, our results display that the expression level of hsa_circ_0005986 was significantly lower in patients who had used the hair dye in the last five years (Figure 2). Several studies have revealed that hair dyes are consist of endocrine‐disrupting combinations and carcinogens which are probably associated with breast cancer. A study by Eberle et al. showed that the risk of BC in black and white women who had used hair permanent dye was 45% and 7% higher, respectively.^45^ Also, Heikkinen et al. have shown that the odds of BC are 23% higher in hair dye users than non‐users.^46^ Therefore, it seems that hair dye components can affect the gene expression pattern through regulatory elements such as circRNAs, but further investigations are needed to clarify the exact association of circRNAs like hsa_circ_0005986 and hair dye in the breast cancer context. Also, we used online bioinformatics tools to evaluate the most relevant genes to BC in hsa_circ_000839/miRNA/mRNA network. The SMAD3, ZEB1, SNAI2, FOXM1, NFKB1, SMAD7, E2F1, ATF3, and hsa‐mir‐1200 had the most interaction among the mRNAs, and miRNA, respectively. There are copious experimental studies that suggest almost all of these genes have a pivotal role in BC development, invasion, and metastasis and the interaction of hsa_circ_000839 with them can augment our hypothesis about its association with BC circumstances.^47^, ^48^, ^49^, ^50^, ^51^, ^52^, ^53^, ^54^, ^55^ Also, bioinformatics construction of the hsa_circ_0005986/miRNA/mRNA network suggests that the GLI1, SMAD4, SP1, and FOXO4, and hsa‐mir‐326, and hsa‐mir‐421 had the most interaction among the mRNAs and miRNA, respectively. In addition, some of these genes have important roles in BC development and prognosis and their interactions with hsa_circ_0005986 make its importance clearer.^47^, ^56^, ^57^, ^58^, ^59^, ^60^

Also, the expression of mRNAs and miRNAs in two networks was evaluated by using TCGA data and Gene Ontology (GO) analysis, and the results of these analyses were shown the significance of mRNAs and miRNAs related to these pathways that this issue provides another evidence for the importance of related circRNAs as potential biomarkers in BC.

Also, in addition to several coding and non‐coding genes which were shown that have an important role in the pathophysiology of BC, evaluation of genes that were involved in the hsa_circ_000839/miRNA/mRNA network revealed that IFNG and IGF1R upregulation and downregulation affect the survival of BC patients, respectively, and this can be another evidence that emphasizes the importance of this circRNA in BC (Figure 7A,B).

## 5 CONCLUSION

In conclusion, a significant decrease of hsa_circ_000839 and hsa_circ_0005986 in tumor tissues compared to adjacent normal tissues support the hypothesis that these circRNAs may function as tumor suppressors in BC. Although our bioinformatics analysis reveals the importance of these two circRNAs and their ceRNA regulatory networks. There is little and controversial experimental evidence about the role of both hsa_circ_000839 and hsa_circ_0005986 in tumors, and more functional studies are needed to confirm the exact roles of these circRNAs.

## CONFLICT OF INTEREST

None.

## Supporting information

Supplementary Material

Supplementary Material

## Data Availability

The data used to support the findings of this study are available from the corresponding author upon request.
