# Supplementary material for: Potential roles of hsa_circ_000839 and hsa_circ_0005986 in breast cancer
Source: J Clin Lab Anal. 2022 Jan 31;36(3):e24263. doi: 10.1002/jcla.24263 (PMC8906031; doi:10.1002/jcla.24263)
Supplement: Supplementary file 1 — Supplementary Material [file JCLA-36-e24263-s001.docx]

| miRNA | mRNA | pearson's R |
| --- | --- | --- |
| hsa-mir-944 | HECW2 | -0.024 |
| hsa-mir-944 | NLK | -0.099 |
| hsa-mir-944 | PTP4A1 | -0.073 |
| hsa-mir-944 | S100PBP | 0.049 |
| hsa-mir-944 | SIAH1 | -0.039 |
| hsa-mir-654-3p | AKT1 | 0.03 |
| hsa-mir-654-3p | CDKN1A | 0.147 |
| hsa-mir-654-3p | EXOG | 0.084 |
| hsa-mir-654-3p | NACC1 | -0.062 |
| hsa-mir-331-3p | ATF3 | -0.133 |
| hsa-mir-331-3p | CTNND1 | 0.136 |
| hsa-mir-331-3p | DOHH | -0.278 |
| hsa-mir-331-3p | E2F1 | 0.116 |
| hsa-mir-331-3p | ERBB2 | -0.017 |
| hsa-mir-331-3p | HOTAIR | -0.031 |
| hsa-mir-331-3p | ING5 | -0.253 |
| hsa-mir-331-3p | NACC1 | -0.08 |
| hsa-mir-331-3p | NRP2 | 0.005 |
| hsa-mir-331-3p | PHLPP1 | 0.059 |
| hsa-mir-409-3p | AKT1 | -0.018 |
| hsa-mir-409-3p | ANG | -0.098 |
| hsa-mir-409-3p | CTNND1 | 0.106 |
| hsa-mir-409-3p | ELF2 | 0.043 |
| hsa-mir-409-3p | FGB | 0.132 |
| hsa-mir-409-3p | FGG | 0.109 |
| hsa-mir-409-3p | FRAT1 | -0.105 |
| hsa-mir-409-3p | GAB1 | 0.115 |
| hsa-mir-409-3p | IFNG | 0.036 |
| hsa-mir-409-3p | MET | 0.186 |
| hsa-mir-409-3p | MGMT | -0.105 |
| hsa-mir-409-3p | NLK | -0.027 |
| hsa-mir-409-3p | PHF10 | -0.028 |
| hsa-mir-409-3p | PASGRP3 | 0.108 |
| hsa-mir-409-3p | ROX | 0.144 |
| hsa-mir-409-3p | RECK | 0.432 |
| hsa-mir-409-3p | RSU1 | 0.172 |
| hsa-mir-409-3p | STAG2 | 0.029 |
| hsa-mir-409-3p | TGFBR2 | 0.34 |
| hsa-mir-409-3p | ZEB1 | 0.437 |
| hsa-mir-590-5p | ATF3 | -0.05 |
| hsa-mir-590-5p | BTG2 | -0.439 |
| hsa-mir-590-5p | CHL1 | -0.225 |
| hsa-mir-590-5p | CREB5 | 0.027 |
| hsa-mir-590-5p | ILF3 | 0.162 |
| hsa-mir-590-5p | OLR1 | -0.042 |
| hsa-mir-590-5p | RB1 | -0.218 |
| hsa-mir-590-5p | RECK | -0.231 |
| hsa-mir-590-5p | SMAD3 | -0.258 |
| hsa-mir-590-5p | SMAD7 | -0.21 |
| hsa-mir-590-5p | TGFB1 | -0.275 |
| hsa-mir-590-5p | TGFBR2 | -0.222 |
| hsa-mir-654-3p | AKT1 | 0.03 |
| hsa-mir-654-3p | CDKN1A | 0.147 |
| hsa-mir-654-3p | EXOG | 0.084 |
