# Supplementary material for: Potential roles of hsa_circ_000839 and hsa_circ_0005986 in breast cancer
Source: J Clin Lab Anal. 2022 Jan 31;36(3):e24263. doi: 10.1002/jcla.24263 (PMC8906031; doi:10.1002/jcla.24263)
Supplement: Supplementary file 2 — Supplementary Material [file JCLA-36-e24263-s002.docx]

| miRNA | mRNA | Pearson's R |
| --- | --- | --- |
| hsa-mir-421 | SIRT3 | -0.122 |
| hsa-mir-421 | CBX7 | -0.157 |
| hsa-mir-421 | RBMXL1 | -0.071 |
| hsa-mir-421 | CASP3 | 0.109 |
| hsa-mir-421 | CDH1 | -0.018 |
| hsa-mir-421 | SMAD4 | -0.141 |
| hsa-mir-421 | FOXO4 | -0.015 |
| hsa-mir-421 | ATM | -0.098 |
| hsa-mir-326 | SMO | -0.123 |
| hsa-mir-326 | PKM | -0.028 |
| hsa-mir-326 | NOB1 | -0.024 |
| hsa-mir-326 | SP1 | 0.054 |
| hsa-mir-326 | FSCN1 | -0.086 |
| hsa-mir-326 | FGF1 | -0.023 |
| hsa-mir-326 | NOTCH1 | -0.044 |
| hsa-mir-326 | NOTCH2 | 0.036 |
| hsa-mir-326 | CD274 | 0.094 |
| hsa-mir-326 | BCL2L1 | -0.018 |
| hsa-mir-326 | GLI1 | -0.022 |
| hsa-mir-326 | HOTAIR | -0.037 |
| hsa-mir-326 | CD9 | -0.072 |
| hsa-mir-326 | MSH3 | 0.062 |
| hsa-mir-188-3p | CBL | 0.112 |
